# Late Stage Cervical Cancer among Confirmed Cervical Cancer Cases in a Tertiary Care Centre: A Descriptive Cross-sectional Study

**DOI:** 10.31729/jnma.6630

**Published:** 2021-07-31

**Authors:** Neebha Ojha, Meena Jha, Eliza Shrestha, Ganesh Dangal

**Affiliations:** 1 Department of Obstetrics and Gynaecology, Tribhuvan University, Institute of Medicine, Kathmandu, Nepal; 2 Paropakar Maternity & Women's Hospital, Kathmandu, Nepal; 3 Department of Obstetrics and Gynaecology, Bhaktapur Cancer Hospital, Kathmandu, Nepal; 4 Department of Obstetrics and Gynaecology, Kathmandu Model Hospital, Kathmandu, Nepal

**Keywords:** *cervical cancer*, *clinical factors*, *demographic factors*, *late-stage*, *Nepal*

## Abstract

**Introduction:**

Cervical cancer is the leading gynaecological cancer in Nepal. Most of the time, it is diagnosed in the late stage with its associated morbidity and mortality. This study aimed to find out the prevalence of late-stage presentation of cervical cancer among confirmed cases of cervical cancer in a tertiary care centre.

**Methods:**

A descriptive cross-sectional study was conducted at a tertiary care centre of Nepal from March 2021 to May 2021 after obtaining ethical clearance from the Institutional Review Board (Reference no.805). A convenient sampling method was used. A descriptive analysis was done of all cases of cervical cancer who were diagnosed within the last 36 months and attended hospital during the study period. The information was collected by interview and hospital record was checked. Analysis was done using Statistical Package for Social Sciences version 20. Point estimate at 95% Confidence Interval was calculated along with frequency and proportion for binary data.

**Results:**

Among the 142 confirmed cervical cancer cases, the prevalence of late-stage presentation of cervical cancer was 93 (65.5%) (57.7-73.3 at 95% Confidence Interval). The mean age at diagnosis was 50.6±10.9 years. More than two-thirds of the women were from outside Kathmandu valley 102 (71.8%) and came from >50km distance. The majority of the women 83 (58.5%) were illiterate.

**Conclusions:**

The study showed that two-thirds of the women presented in advanced stage and the factors leading to the late stage. This highlights the fact, that the focus should be on the provision of organized screening programs and early diagnosis and treatment of cervical cancer.

## INTRODUCTION

Globally cervical cancer is the fourth most frequent cancer in females and represents 6.6% of all female cancers.^1^ However, in Nepal it is the most common cancer in females.^2^ More than 90% of cervical cancers occur in developing countries.^3^ Approximately 84% of all cervical cancers and 88% of all deaths caused by cervical cancer occurred in lower-resource countries.^1^

A study in two cancer hospitals of Nepal showed 80.9% of cervical cancer had late-stage presentation.^4^ Factors such as race, age at diagnosis, marital status,^5^ educational status,^6^ places of residence,^7^ number of children,^8^ and screening practices^9^ have been associated with the late-stage presentation of cervical cancer. Knowing the burden of late-stage presentation of cervical cancer may provide a base on which appropriate steps for prevention and treatment can be planned.

This study aimed to find out the prevalence of late-stage presentation of cervical cancer among confirmed cases of cervical cancer in a tertiary care centre.

## METHODS

This is a descriptive cross-sectional study conducted at Bhaktapur Cancer Hospital, Nepal from March 2021 to May 2021. Ethical approval was taken from the Institutional Review Board (Ref no.805). Convenient sampling method was used. All cases of cervical cancer diagnosed within the last 36 months and reporting to the out-patient department or admitted in the ward on surgical treatment, radiotherapy, chemoradiation, chemotherapy, palliative treatment were taken for the study after consent. Patient's whose diagnosis was not clear about the origin of the tumour were excluded from the study.

Sample size was estimated using the following formula:

n = Z^2^ × p × q / e^2^

  = (1.96)^2^ × 0.81 × (1-0.81) / (0.07)^2^

  = 121

Where,

n = minimum required sample size
Z = 1.96 at 95% Confidence Interval
p = prevalence from previous study, 0.81^4^
q = 1-p
e = margin of error,7%

Adding the 10% non-response rate the final sample size was 133. However, the total number of cases enrolled in the study was 142.

The information was collected by interview regarding the demographic factors such as age at diagnosis, menopausal status at diagnosis, marital status, parity, place of residence, distance from the health facility, education level and clinical factors such as initial symptoms of cervical cancer, history of screening for cervical cancer, history of smoking and health-seeking interval. The record was checked regarding the type of tumour and stage at diagnosis of cervical cancer. Details of the treatment received were noted. The stage at diagnosis was divided as early-stage for Stages I to IIA and late-stage for Stages IIB to IV.

The collected data were entered and analysed in SPSS (Statistical Package for Social Sciences) version 20. Descriptive statistics were used and the categorical variables were interpreted by frequencies and percentages. Continuous variables were summarized into means with standard deviations.

## RESULTS

Out of the 142 cases of diagnosed cervical cancer, 93 (65.5%) (57.7-73.3 at 95% Confidence Interval) were of late-stage (IIB-IV) cervical cancer. The mean age at diagnosis of cervical cancer was 50.6±10.9 years ranging from 30 to 78 years. Most of the women were above 50 years 74 (52.1%) and postmenopausal 75 (52.8%) at diagnosis. The mean parity was 3.6±1.8. More than two-thirds of the women were from outside Kathmandu valley 102 (71.8%) and came from >50km distance. The majority of the women 83 (58.5%) were illiterate (Table 1).

**Table 1:** Demographic characteristics of cervical cancer cases (n= 142).

Demographic Factors	n (%)
**Age at diagnosis (years)**	
<40	23 (16.2)
40-49	45 (31.7)
≥50	74 (52.1)
**Menopausal at diagnosis**	
Yes	75 (52.8)
No	67 (47.2)
**Marital status**	
Married	116 (81.7)
Unmarried	0
Separated	4 (2.8)
Divorced	33 (15.2)
Widowed	0
**Parity**	
1	3 (2.1)
2-4	107 (71.8)
≥5	32 (22.5)
**Place of residence**	
Kathmandu valley	40 (28.2)
Outside Kathmandu	102 (71.8)
**Distance from a health facility (km)**	
<50	42 (29.6)
50-100	26 (18.3)
>100	74 (52.1)
**Education level**	
Illiterate	83 (58.5)
Primary (class 1-5)	39 (27.5)
Secondary (6-12)	20 (14.1)
Higher secondary and above	0 (0)

Among the clinical factors, abnormal vaginal bleeding was the most common initial symptom present in 117 (82.4%) women. Among them, 36 (25.4%) had intermenstrual and 23 (16.2%) had post-coital bleeding. Among the 75 postmenopausal ladies, 58 (77.3%) had postmenopausal bleeding. Squamous cell carcinoma was present in 129 (90.8%) women. Four women (2.8%) had no symptoms at all. Only 16 (11.3%) had a history of screening. Fifty nine (41.5%) had delayed health-seeking interval (≥3months) after initial symptoms (Table 2). On further questioning, the most common reason for the delayed health-seeking interval was, not taking it as a serious problem 32 (54.2%), followed by financial reason 11 (18.7%) and lockdown due to Covid19 pandemic 11 (18.7%), few sought traditional healers 3 (5.1%) or had fear of diagnosis in 2 (3.4%) women.

**Table 2:** Clinical characteristics cases of cervical cancer (n= 142).

Clinical Factors	n (%)
**Initial symptoms** ^*^	
Abnormal vaginal bleeding	117 (82.4)
Abnormal vaginal discharge	91 (64.1)
Pain lower abdomen/pelvic pain	56 (39.4)
None	4 (2.8)
**History of screening for cervical cancer**	
Yes	16 (11.3)
No	122 (85.9)
Don't know	4 (2.8)
**History of smoking**	
Yes	49 (35.5)
No	93 (65.5)
**Health seeking interval**	
<3 months	83 (58.5)
≥3 months	59 (41.5)
**Histopathology report**	
Squamous cell	129 (90.8)
Adenocarcinoma	13 (9.2)

* May have more than one response

Table 3 shows the distribution of late-stage presentation of cervical cancer in different demographic factors. According to the education level, 31 (52.5%) literates had late-stage, while among the illiterate women 62 (74.7%) were in late-stage at presentation (Table 3).

**Table 3:** Distribution of late-stage presentation of cervical cancer among different demographic factors (n=142).

Demographic factors	Late-stage at diagnosis	
	Yes n (%)	No n (%)
**Age at diagnosis (years)**		
<50	42 (61.8)	26 (38.2)
≥50	51 (68.1)	23 (31.9)
**Menopausal at diagnosis**		
No	42 (62.7)	25 (37.3)
Yes	52 (68.0)	24 (32.0)
**Marital status**		
Married	75 (64.1)	42 (35.9)
Separated/Widowed	18 (72.0)	7 (28.0)
**Parity**		
≤4	68 (62.4)	41 (37.6)
>4	25 (75.8)	8 (24.2)
**Place of residence**		
Kathmandu valley	22 (55.0)	18 (45.0)
Outside Kathmandu	71 (69.6)	31 (30.4)
**Distance from a health facility (Km)**		
<50	24 (57.1)	18 (42.9)
≥50	69 (69.0)	31(31.0)
**Education level**		
Literate	31(52.5)	28 (47.5)
Illiterate	62 (74.7)	21(25.3)

The distribution of late-stage presentation of cervical cancer in different clinical factors is shown (Table 4).

**Table 4:** Distribution of late-stage presentation of cervical cancer among different clinical factors.

Clinical factors	Late-stage at diagnosis	
	Yes n (%)	No n (%)
**The initial symptom of Abnormal vaginal bleeding (n = 142)**		
No	12 (48.0)	13 (52.0)
Yes	81 (66.4)	36 (30.8)
**Postmenopausal bleeding (n = 75)**		
No	10 (55.6)	8 (44.4)
Yes	39 (70.9)	16 (29.1)
**History of screening for cervical cancer (n = 138)**		
Yes	8 (50.0)	8 (50.0)
No	81 (66.4)	41 (33.6)
**History of smoking (n = 142)**		
No	59 (63.4)	34 (36.6)
Yes	34 (69.4)	15 (30.6)
**Health seeking interval (n = 142)**		
<3 months	45 (54.2)	38 (45.8)
≥3 months	48 (81.4)	11 (18.6)
**Histology of tumour (n = 142)**		
Adenocarcinoma	5 (38.5)	8 (61.5)
Squamous carcinoma	88 (68.2)	41 (31.8)

## DISCUSSION

This study was conducted in a hospital in Kathmandu, Nepal and explored the late-stage presentation of cervical cancer. Of the total cervical cancer cases, 93 (65.5%) women presented in late-stage. It is similar to most of the studies done in developing countries, which shows the majority of the cervical cancer cases diagnosed, were in the late-stage ranging from 65 to 80% as compared to the early stage.^4,8,10^ Mlange, et al. found among 202 patients confirmed with cervical cancer, the prevalence of late-stage cervical cancer was 63.9%.^11^ Studies have shown that late-stage at diagnosis is correlated with lower survival rates in cervical cancer patients.^12^ Approximately 84% of all cervical cancers and 88% of all deaths caused by cervical cancer occurred in lower-resource countries, by contrast, in highest-resource countries, the cumulative rate of cervical cancer and mortality were two to four times lower than those in lower resource countries.^1^

The mean age of the women at diagnosis was 50.6±10.9) years. It is similar to other studies where the mean age of the women presenting to the hospital at diagnosis of cervical cancer ranged from 50-53 years.^4,11,13,14^ Higher age is associated with advanced-stage cervical cancer. The proportion of advanced stage in new invasive cervical cancer was 30% among women aged <50 years and 52% among women aged ≥50 years.^15^ However, in the present study, older age is not associated with the late-stage presentation of cervical cancer as in other studies.^4,10,11^

This study, among the socio-demographic characteristics of the patients, showed that illiterate women were more likely to have late-stage (illiterate 74.7% vs literate 52.5%). Studies from Uganda,^8^ Iran,^6^ Nepal,^4^ suggest that delayed diagnosis of cervical cancer was higher in women with lower-level education. The lower level of education may be indirectly related to lower socioeconomic status, ignorance about the preventive measures as screening which can adversely affect in delay in seeking healthcare.

A systematic review of research on the association between marital status and stage at diagnosis of different types of cancer showed a significant association between marital status and stage at diagnosis, with a positive effect of marriage on the cancer being diagnosed in the earlier stage.^16^ Married women tend to have better health-seeking behaviour.^17^ In a study from Morocco, women who were not married were more likely to present in late-stage cancer.^7^ In the present study, 116 (81.7%) of the women were married and those separated/widows were more likely to have a late-stage diagnosis in comparison to married women (70.0% vs 64.1% respectively).

Place of residence or distance from health facility plays an important factor for earlier presentation. Women living at a further distance were more in late-stage it. Women who live more than 100 km from the location of cancer diagnosis site were four times more likely to present late (OR = 4.51; 95% CI: 1.35-1 5.11 ),^7^ and women living in the rural community were more likely to have delayed diagnosis.^14,18^

The present study showed only 16 (11.3%) had a history of screening for cervical cancer. Among the screened women 50% had late-stage diagnosis while in an unscreened group it was 66.4%, however, the number of cases was quite low. The health delivery system of the country is complex as screening as well as early detection and treatment facilities are concentrated in the major cities and especially in the Kathmandu valley. Studies from national household surveys and communities in Nepal show low knowledge and practice regarding cervical cancer screening.^19,20^ Previously unscreened cases of cervical cancer were nearly four times more likely to present late, compared to those who had been screened previously.^10^ Factors that contribute to the late diagnosis are inadequate knowledge of cervical cancer, lack of screening, and poor diagnostic procedure and treatment among health care providers.^21^

The most common initial symptom was abnormal vaginal bleeding present in 82.4% of cases. The symptom may have prompted the women to seek healthcare but by the time the women become symptomatic she may be in late-stage, which was higher in a late-stage presentation in the present study. A similar finding was seen in another study from Nepal,^4^ but not in other study.^10^

Delayed health-seeking behaviour has been adversely associated with the late-stage presentation of cervical cancer. Reasons for the delayed presentation were, not taking the symptoms as being serious (54.2%), financial reason (18.7%), or lockdown due to Covid19 pandemic in 18.7%. It is intricately associated with the educational status, socioeconomic status, distance to the healthcare centre and knowledge related to cervical cancer.^13^ In our study, 59 (41.5%) had sought healthcare after three months of initial symptoms, who were more likely to present in late-stage (81.2% vs 54.2%) as compared to those who presented earlier. It is similar to another study where longer health-seeking intervals were associated with more advanced cancer stages at pathological diagnosis.^18^

In the present study, squamous cell histology was present in 90.8% of cases. Squamous cell carcinoma accounts for three fourth of cervical cancer. SEER data from the USA suggest an increasing prevalence of adenocarcinoma of the cervix with a poorer prognosis, which could be due to the inefficiency of conventional screening for these tumours.^12^ Cervical cancer predominantly coming in late-stage could be due to women coming only after being symptomatic, rather than during screening program.

The sample size is not very large and there could have been memory bias during the interview. The present study represented many districts of Nepal as it was done in a tertiary care referral cancer hospital where women from all over Nepal come for treatment.

## CONCLUSIONS

The study provides information on the prevalence of late-stage cervical cancer. The late-stage presentation of cervical cancer was almost the same in comparison to a similar study done in similar settings. It was found in higher proportion in older and postmenopausal women, higher parity, living outside Kathmandu valley, illiterate. Having abnormal vaginal bleeding, lack of screening delayed health-seeking intervals all led to the late-stage presentation of cervical cancer. As two-third of the women presented in an advanced stage, provision of an organized screening program and diagnosis and treatment of cervical cancer in early-stage would ultimately improve the survival of the women.
